# Isolation and characterization of three novel *Acinetobacter baumannii* phages from Beninese hospital wastewater

**DOI:** 10.1007/s00705-023-05845-z

**Published:** 2023-08-13

**Authors:** Anna Kolsi, Kaisa Haukka, Victorien Dougnon, Alidehou Jerrold Agbankpè, Kafayath Fabiyi, Marko Virta, Mikael Skurnik, Anu Kantele, Saija Kiljunen

**Affiliations:** 1 Human Microbiome Research Program, Research Programs Unit, Faculty of Medicine, University of Helsinki, Helsinki, Finland; 2 Department of Microbiology, University of Helsinki, Helsinki, Finland; 3 Research Unit in Applied Microbiology and Pharmacology of natural substances, Polytechnic School of Abomey-Calavi, University of Abomey-Calavi, Abomey Calavi, Benin; 4 Division of Clinical Microbiology, HUS Diagnostic Center, Hospital District of Helsinki and Uusimaa, Helsinki, Finland; 5 Meilahti Vaccine Research Center MeVac, Infectious Diseases, Helsinki University, Hospital District of Helsinki and Uusimaa, Helsinki, Finland

## Abstract

*Acinetobacter baumannii* is an opportunistic pathogen that is mostly associated with hospital-acquired infections. The rapid emergence of multi- and pan-drug-resistant *Acinetobacter* strains poses an increasing challenge in hospitals. Phage therapy offers one treatment option for infections caused by *A. baumannii*. We isolated three phages from Beninese hospital wastewater – fBenAci001, fBenAci002, and fBenAci003 – that infected clinical *A. baumannii* strains from Finnish patients. Phylogenetic analysis showed that these phages resemble phages of the genus *Friunavirus*, family *Autographiviridae.* The isolated phages meet the requirements set for phages used for phage therapy. However, they were found to have a narrow host range, which may limit their therapeutic use.

**Supplementary Information:**

The online version contains supplementary material available at 10.1007/s00705-023-05845-z.

*Acinetobacter baumannii* is a Gram-negative nosocomial opportunistic pathogen. It is associated with a wide spectrum of hospital-acquired infections, such as pneumonia, bloodstream infections, urinary tract infections, and wound infections [1]. The World Health Organization published a global priority list of multidrug-resistant (MDR) bacteria in 2017 in which carbapenem-resistant *A. baumannii* appeared in the first priority group [2]. *A. baumannii* is also a part of the ESKAPE (*Enterococcus faecium*, *Staphylococcus aureus*, *Klebsiella pneumoniae*, *A. baumannii*, *Pseudomonas aeruginosa*, and *Enterobacter* species) pathogen group with exceptionally high resistance towards antibiotics [3]. *A. baumannii* has numerous antibiotic resistance mechanisms, which is reflected by the fact that the proportion of MDR strains reached 64% in 2014 [4]. Although *A. baumannii* causes a minor proportion of all infections associated with Gram-negative pathogens, mortality rates as high as 40–50% have been associated with these infections in intensive care units [4].

One promising method to treat MDR *A. baumannii* infections is phage therapy (PT), which utilizes bacteriophages (phages), which are viruses that infect bacteria. Phages are highly specific and only infect their target bacteria, and they do not affect the patient’s normal microbiota or human cells. Other advantages of phage therapy are, for example, their self-dosing capability, effective biofilm clearance, and low environmental impact. One of the disadvantages of PT is its narrow host range, which makes it difficult to find a suitable phage [5]. In addition, every phage used for phage therapy has to be characterized and found to be free of genes that encode bacterial toxins or are associated with antibiotic resistance, transposable elements, or a lysogenic life cycle [6].

Phage therapy has been shown to successfully cure *A. baumannii* infections when antibiotic medications have failed [4, 7]. Phages infecting *A. baumannii* have previously been isolated from various sources, most typically from hospital wastewater outside of Western countries [8–11]. Since antibiotic resistance is a major problem worldwide, effective therapeutic phages are needed globally.

Here, we describe the isolation and characterization of three phages from Beninese hospital wastewater. All bacterial strains used in this study were clinical *Acinetobacter* isolates (Table 1), most of which were collected from Finnish patients, but with unknown geographical origins. Samples for phage isolation included 34 wastewater samples that were collected during November and December 2019 in Benin from hospitals located in the adjacent cities Cotonou and Abomey-Calavi [12]. None of the hospitals were connected to a sewer system, and the samples were obtained from septic tanks or sumps. In most cases, the toilet water was not directed into these sumps. To remove debris and bacteria, the samples were filtered through a 0.2-µm polycarbonate filter (Whatman™, GE Healthcare Life Sciences), and the filtrates were transported to Finland.

**Table 1:** *Acinetobacter* strains used in the study

Species	Strain no.	Resistance	Origin	Growth medium	Sensitive to	Reference
*Acinetobacter baumannii*	5542*	MDR	Not known	LB	fBenAci001**	HUSLAB
	5568*		Blood	LB		HUSLAB
	5570*		Blood	LB		HUSLAB
	5596*	MDR	Bronchus	LB	fBenAci003	HUSLAB
	5597*	MDR	Rectum	LB		HUSLAB
	5706*		Tooth removal hole	LB		HUSLAB
	5707*	MDR	Hip surgical wound	LB	fBenAci002**	HUSLAB
	5729*	MDR	Rectum	LB		HUSLAB
	5730*	MDR	Rectum	LB		HUSLAB
	5731*		Middle ear	LB		HUSLAB
	5907*		Trachea	LB		HUSLAB
	5910*	MDR	Skin	LB	fBenAci003**	HUSLAB
	5911*	MDR	Rectal mucus	LB		HUSLAB
	5919*	MDR	Rectal mucus	LB		HUSLAB
	5920*	MDR	Not known	LB	fBenAci002	HUSLAB
	5923*	MDR	Wound pus	LB		HUSLAB
	5924*		Blood	LB		HUSLAB
	5933*		Urine	LB		HUSLAB
	5934*		Not known	LB		HUSLAB
	6594*	MDR	Bronchus	LB		HUSLAB
	6597*	MDR	Not known	LB		DSM 106838
	6898*	MDR	Sacral chronic wound	LB		HUSLAB
	6906*		Trachea	LB		Germany
*Acinetobacter calcoaceticus*	5922		Blood	LB		HUSLAB
	5935		Not known	LB		HUSLAB
*Acinetobacter junii*	5567	MDR	Stool	BHI		HUSLAB
*Acinetobacter lwoffii*	5912		Blood	LB		HUSLAB
	5921		Blood	LB		HUSLAB
	5928		Blood	LB		HUSLAB
*Acinetobacter nosocomialis*	5901		Foot surgical wound	LB		HUSLAB
	5904		Peritoneal abscess	LB		HUSLAB
	5929		Urine	LB		HUSLAB
*Acinetobacter pittii*	5565		Blood	LB		HUSLAB
	5566		Blood	LB		HUSLAB
	5573		Blood	LB		HUSLAB
	5673		Blood	LB		HUSLAB
	5674		Wound pus	LB		HUSLAB
	5728		Wound pus	LB		HUSLAB
	5902		Not known	LB		HUSLAB
	5903		Not known	LB		HUSLAB
	5905		Leg wound pus	LB		HUSLAB
	5906		Urine	LB		HUSLAB
	5908		Cerebrospinal fluid	LB		HUSLAB
	5909		Blood	LB		HUSLAB
	5914		Blood	LB		HUSLAB
	5917		Blood	LB		HUSLAB
	5918		Blood	LB		HUSLAB
	5925		Blood	LB		HUSLAB
	5930		Urine	LB		HUSLAB
	5931		Urine	LB		HUSLAB
*Acinetobacter radioresistens*	5915		Blood	LB		HUSLAB
	5916		Blood	LB		HUSLAB
*Acinetobacter ursingii*	5569		Not known	BHI		HUSLAB
	5572		Not known	BHI		HUSLAB
	5913		Blood	BHI		HUSLAB
	5927		Skin	LB		HUSLAB
	5932		Urine	BHI		HUSLAB

*Used as an enrichment host

**Original isolation host of the phage

MDR, multi-drug resistant; LB, lysogeny broth; BHI, brain heart infusion; HUSLAB, Division of Clinical Microbiology, HUS Diagnostic Center, the Hospital District of Helsinki and Uusimaa, Finland

Bacterial and phage incubations were conducted in lysogeny broth (LB) [13] unless otherwise stated. For solid and semisolid media, LB was supplemented with 1.5% and 0.4% agar, respectively. All bacterial and phage cultures were maintained at 37^o^C using standard methods [13]. A total of 23 *A. baumannii* strains (Table 1) were used for phage isolation by a previously described method [14]. Wastewater samples were pooled into four cocktails, and each cocktail was tested against individual strains. Each phage that was obtained was further purified by three rounds of plaque purification [13]. To determine its host range, each phage was tested against 57 *Acinetobacter* strains (Table 1) by the liquid culture method, using a Bioscreen C plate reader [15].

Phage DNA was isolated from a raw high-titer lysate (10^10^ plaque-forming units PFU/ml) using phenol-chloroform extraction and ethanol precipitation [13]. DNA samples were sequenced by the 150-bp paired-end protocol on an Illumina HiSeq platform at Novogene (https://en.novogene.com/). For *de novo* assembly of the phage genome sequences, a 50,000-read subset was made from both forward and reverse sequence files, using Chipster v4 [16]. Genome sequences were assembled using the A5-miseq integrated pipeline for the *de novo* assembly of microbial genomes [17]. BLASTn 2.10.1+ [18] was used to identify phage genome sequences among the resulting contigs. PhageTerm [19] was used to predict the physical ends of the phage genomes. The genome sequences were trimmed and organized manually based on the PhageTerm results. Assemblies were confirmed using Geneious Prime 2020.1.2 (https://www.geneious.com/) by mapping all original reads (7,913,416, 7,097,864, and 5,793,626 reads for fBenAci001, fBenAci002, and fBenAci003, respectively) back to the *de novo* assemblies. Preliminary annotations were made using RAST 2.0 [20–22], after which the annotations were checked and edited manually using Artemis 18.1.0 [23], BLASTp 2.10.1 [24], and HHpred [25]. The sequences were examined for the presence of tRNA genes using Aragorn v1.2.38 [26], VirulenceFinder 2.0 [27] was used to screen for genes coding for bacterial toxins, and ResFinder 4.0 [28] and CARD 3.0.7 [29] were used to screen for antibiotic resistance genes. All of the bioinformatic tools were applied using standard parameters.

Genome sequences were aligned using the Geneious Prime alignment tool with standard settings. Based on the alignment, the least similar protein that the phages had in common (tailspike), and one conserved structural protein (capsid protein) were selected for phylogenetic analysis. The closest relatives of the phages at the whole-genome level were identified using BLASTn, and matches with over 90% query coverage (Supplementary Table S1) were used for the construction of phylogenetic trees and a genome similarity heat map. The predicted amino acid sequences of the tailspike and capsid proteins were identified in the selected genome sequences and re-annotated, if needed.

A genome-wide phylogenetic tree was constructed using VICTOR (Virus Classification and Tree Building Online Resource) [30] using Genome-BLAST Distance Phylogeny [31] with the standard settings recommended for the prokaryotic viruses [30]. The resulting intergenomic distances were used to infer a balanced minimum evolution tree with branch support via FASTME including SPR postprocessing [32]. Branch support was inferred from 100 pseudo-bootstrap replicates. A tree was rooted at the midpoint [33] and visualized with ggtree [34]. Taxon boundaries at the species, genus, and family level were estimated using the OPTSIL program, the recommended clustering thresholds [30], and an F value (fraction of links required for cluster fusion) of 0.5 [35]. Phylogenetic trees for protein-level connections were built using the GGDC web server [31], which uses the DSMZ phylogenomics pipeline [35] adapted to single genes. A genome similarity heat map was generated using the VirClust WEB server at Virus Intergenomic Distance Calculator VIRIDIC [36].

Phages fBenAci001, fBenAci002, and fBenAci003 were isolated using *A. baumannii* strains #5542, #5707, and #5910, respectively. All three phages originated from the same wastewater pool, and each of them produced clear plaques with slightly different sizes, with fBenAci001 and fBenAci002 producing a halo (Supplementary Fig. S1). Under optimal conditions, all three phages gave a high titer (10^9^-10^10^ PFU/ml) in their isolation hosts. All three phages were found to have a very narrow host range. Phage fBenAci001 infected only its original isolation host, and phages fBenAci002 and fBenAci003 were able to infect one additional *A. baumannii* strain each (Table 1).

An overview of the phage genomes is presented in Table 2. The genome sequences of phages fBenAci001, fBenAci002, and fBenAci003 are available in the GenBank database under the accession numbers MW056501, MW056502, and MW056503, respectively. All phages had approximately 41-kbp genomes and a GC content of 39.2. All predicted coding sequences (CDSs) were in the forward orientation. Direct terminal repeats with lengths of 368–394 bp were identified using PhageTerm. No genes encoding tRNAs or bacterial toxins or genes associated with antibiotic resistance, transposable elements, or a temperate life cycle were identified in the genomes.

**Table 2:** Overview of the phages

	fBenAci001	fBenAci002	fBenAci002
Accession number	MW056501	MW056502	MW056503
Family	*Autographiviridae*	*Autographiviridae*	*Autographiviridae*
Genus	*Friunavirus*	*Friunavirus*	*Friunavirus*
Genome size (bp)	40,535	41,953	40,556
GC content (%)	39.2	39.2	39.2
Length of direct terminal repeats (bp)	394	375	368
tRNA genes	None	None	None
Bacterial toxin genes	None	None	None
Antibiotic resistance genes	None	None	None
Identity to fBenAci001	100%	82.2%	79.5%
Identity to fBenAci002	82.2%	100%	80.8%
Identity to fBenAci003	79.5%	80.8%	100%

A comparison of the phage genomes using VIRDIC showed that the sequence identity of all three phages to their closest relatives was between 80% and 91% (Supplementary Fig. S2). A Genome-BLAST Distance Phylogeny tree using the distance formula D0 is presented in Fig. 1A. The OPTSIL clustering yielded 21 species clusters. The tree revealed that phages fBenAci001, fBenAci002, and fBenAci003 are closely related to each other and resemble phages of the genus *Friunavirus*, family *Autographiviridae*. Each phage represents a novel, separate species. The deduced amino acid sequences of the predicted tail spike proteins (Fig. 1B) and capsid proteins (Fig. 1C) were used to build protein-level phylogenetic trees, using the GGDC web server. When comparing the protein-level phylogenetic trees with the tree based on whole genome sequences, a mosaic-like pattern was seen instead of a clear correlation.

**Fig. 1:**
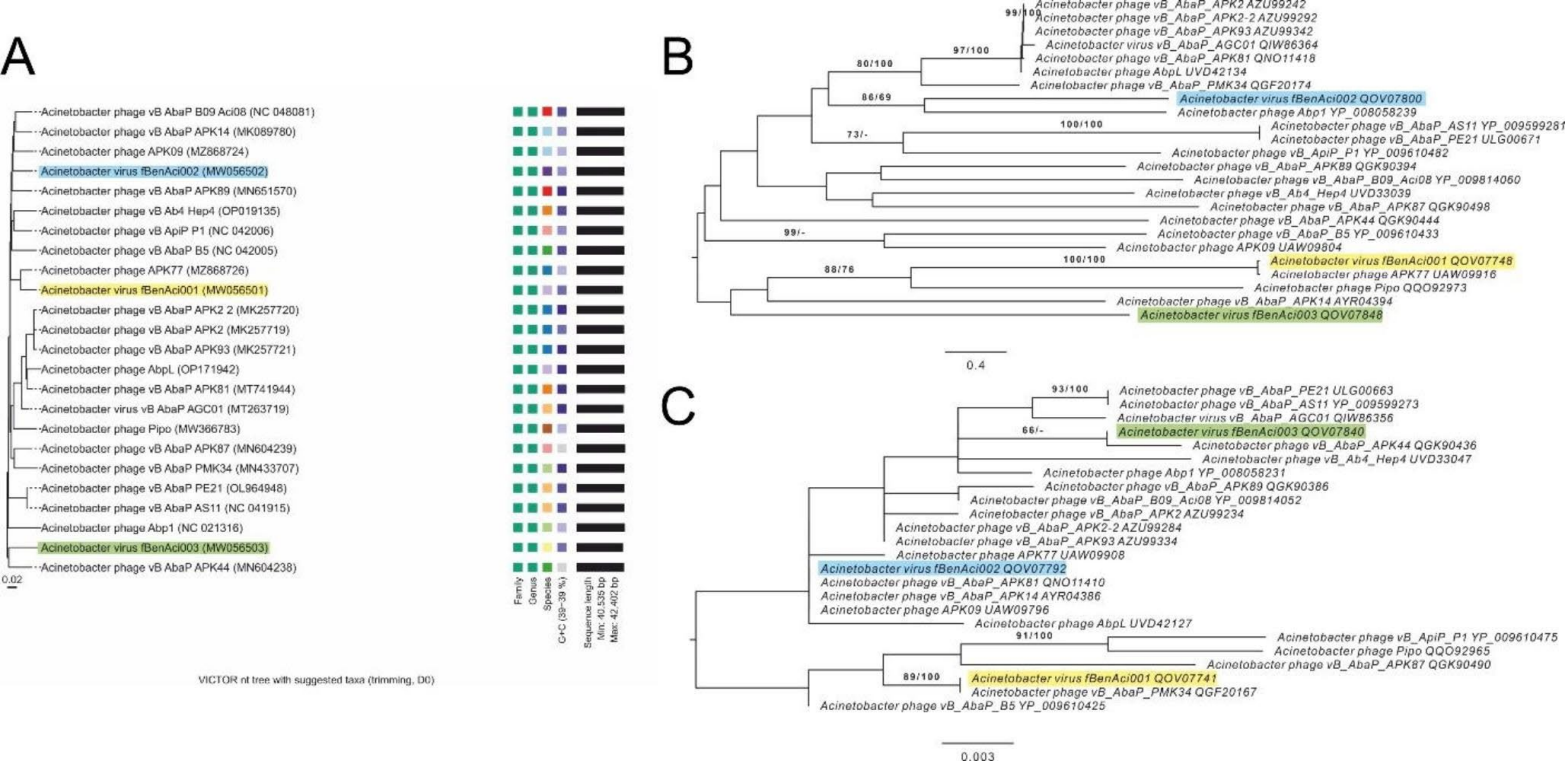
Phylogenetic trees of the phages fBenAci001 (yellow), fBenAci002 (blue), and fBenAci003 (green) and their 21 closest relatives. (**A**) Genome-BLAST Distance Phylogeny tree using the distance formula D0, constructed using VICTOR. The colors of the symbols in the “species” column indicate whether phages belong to the same or different species within the same family and genus. (**B**) Phylogenetic tree based on the tailspike protein, calculated using the GGDC web server. (**C**) Phylogenetic tree based on the capsid protein, calculated using the GGDC web server. CorelDRAW was used to create the panel

To conclude, three phages infecting clinical *A. baumannii* strains from Finnish patients were isolated from Beninese water samples. Phages fBenAci001, fBenAci002, and fBenAci003 were identified as three novel phages related to members of the genus *Friunavirus* of the family *Autographiviridae*. The phages were found to have a very narrow host range, which is a typical feature of *A. baumannii* phages [37–39]. Based on previous studies, the *Friunavirus* phages have varying capability to infect *A. baumannii* strains, infecting 3–50% of the strains tested. In most of the studies that showed a broad (over 30%) host range, all of the strains were isolated from the same hospital [11, 40] or from an otherwise limited area [41]. This might suggest that the bacterial strains tested in those studies had at least a partially clonal origin. When phage infectivity was tested against strains from diverse origins, the phages typically infected only a few strains [39] or just the isolation host [8].

As there were no known genes associated with a temperate life cycle, transposable elements, antibiotic resistance, or bacterial virulence in their genomes, phages fBenAci001, fBenAci002, and fBenAci003 meet the current safety requirements for therapeutic phages. However, it is worth noting that the functions of around half of the predicted gene products were unidentified. Other phages of the genus *Friunavirus* have been used successfully in phage therapy trials [41, 42], supporting the suitability of the phages characterized in this study for phage therapy. However, their narrow host range may limit the usefulness of these phages in therapeutic applications.

The phages characterized in this study were isolated from hospital wastewater from Benin, confirming an earlier finding by Essoh et al. [9] that wastewater from Africa is a good source for *A. baumannii* phages. In our experience, isolating *A. baumannii* phages from Finnish wastewater is very challenging, and water samples from other locations are therefore important resources for these phages. In Finland, *Acinetobacter* infections are often linked to travel. A Finnish study analyzing the origin of MDR *Acinetobacter* colonization in hospitalized patients transferred to Finland from other countries showed that most MDR *Acinetobacter* originated from Asia, North Africa, or the Middle East [43]. Notably, there was no MDR *Acinetobacter* transferred from sub-Saharan Africa between 2010 and 2019, presumably due to the low numbers of travelers to the region. However, in a report from 2019, the all-age death rate attributable to antimicrobial resistance was the highest in western sub-Saharan Africa [44]. For this reason, it is important to isolate new phages from this area to help fight against resistant infections.

## Electronic Supplementary Material

Below is the link to the electronic supplementary material

Supplementary Material 1

## Data Availability

The genomic sequences of phages fBenAci001, fBenAci002, and fBenAci003 have been submitted to the GenBank database under the accession numbers MW056501, MW056502, and MW056503, respectively. All data generated or analyzed in this study are included in this published article and its supplementary files.
