# Supplementary material for: Isolation and characterization of three novel Acinetobacter baumannii phages from Beninese hospital wastewater
Source: Arch Virol. 2023 Aug 13;168(9):228. doi: 10.1007/s00705-023-05845-z (PMC10423700; doi:10.1007/s00705-023-05845-z)
Supplement: Supplementary file 1 — Supplementary Material 1 [file 705_2023_5845_MOESM1_ESM.docx]

**Supplementary data**

*Title*

**Isolation and characterization of three novel *Acinetobacter baumannii* phages from Beninese hospital wastewater**

Anna Kolsi^1,2^, Kaisa Haukka^1,2^, Victorien Dougnon^3^, Alidehou Jerrold Agbankpè^3^, Kafayath Fabiyi^3^, Marko Virta^2^, Mikael Skurnik^1,4^, Anu Kantele^1,5^, Saija Kiljunen^1,4^*

^1^Human Microbiome Research Program, Research Programs Unit, Medicum, Faculty of Medicine, University of Helsinki, Finland

^2^Department of Microbiology, University of Helsinki, Finland

^3^Research Unit in Applied Microbiology and Pharmacology of natural substances, Polytechnic School of Abomey-Calavi, University of Abomey-Calavi, Benin

^4^Division of Clinical Microbiology, HUS Diagnostic Center, Hospital District of Helsinki and Uusimaa, Helsinki, Finland

^5^Meilahti Vaccine Research Center MeVac, Infectious Diseases, Helsinki University, Hospital District of Helsinki and Uusimaa, Helsinki, Finland

*Author for correspondence: saija.kiljunen@helsinki.fi

Table of content

**Supplementary Fig.1.** Double layer overlay plates after 16 hours of incubation on 0.4% Lysogeny broth soft agar and Luria broth plates.

**Supplementary table 1.** Accession numbers and protein ids for phylogenetic analysis.

**Supplementary Fig.2.** Similarity heatmap of phage genomes used for phylogeny analysis.


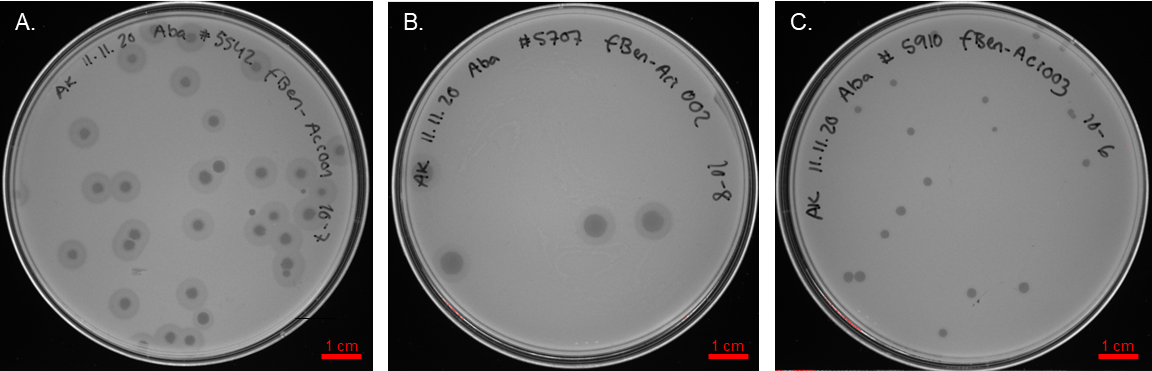


**Supplementary Fig.1.** Double layer overlay plates after 16 hours of incubation on 0.4% Lysogeny broth soft agar and Lysogeny broth plates. A. fBenAci001. B. fBenAci002. C. fBenAci003.

**Supplementary table 1.** Accession numbers and protein ids for phylogenetic analysis.

| **Phage** | **Accession** | **Tailspike protein** | **Capsid protein** | **Note** |
| --- | --- | --- | --- | --- |
| Acinetobacter virus fBenAci001 | [MW056501.1](https://www.ncbi.nlm.nih.gov/nucleotide/MW056501.1?report=genbank&log$=nucltop&blast_rank=1&RID=X77ND6AR016) | [QOV07748.1](https://www.ncbi.nlm.nih.gov/protein/1925873496) | [QOV07741.1](https://www.ncbi.nlm.nih.gov/protein/1925873489) |  |
| Acinetobacter virus fBenAci002 | [MW056502.1](https://www.ncbi.nlm.nih.gov/nucleotide/MW056502.1?report=genbank&log$=nucltop&blast_rank=1&RID=X79P9D9H013) | [QOV07800.1](https://www.ncbi.nlm.nih.gov/protein/1925873732) | [QOV07792.1](https://www.ncbi.nlm.nih.gov/protein/1925873724) |  |
| Acinetobacter virus fBenAci003 | [MW056503.1](https://www.ncbi.nlm.nih.gov/nucleotide/MW056503.1?report=genbank&log$=nucltop&blast_rank=1&RID=X7ATB8JA016) | [QOV07848.1](https://www.ncbi.nlm.nih.gov/protein/1925873978) | [QOV07840.1](https://www.ncbi.nlm.nih.gov/protein/1925873970) |  |
| Acinetobacter phage vB_AbaP_APK14 | [MK089780.1](https://www.ncbi.nlm.nih.gov/nucleotide/MK089780.1?report=genbank&log$=nucltop&blast_rank=11&RID=X77ND6AR016) | [AYR04394.1](https://www.ncbi.nlm.nih.gov/protein/1509794188) | [AYR04386.1](https://www.ncbi.nlm.nih.gov/protein/1509794180) |  |
| Acinetobacter phage vB_AbaP_APK2 | [MK257719.1](https://www.ncbi.nlm.nih.gov/nucleotide/MK257719.1?report=genbank&log$=nucltop&blast_rank=9&RID=X77ND6AR016) | [AZU99242.1](https://www.ncbi.nlm.nih.gov/protein/1549120150) | [AZU99234.1](https://www.ncbi.nlm.nih.gov/protein/1549120142) |  |
| Acinetobacter phage vB_AbaP_APK2-2 | [MK257720.1](https://www.ncbi.nlm.nih.gov/nucleotide/MK257720.1?report=genbank&log$=nucltop&blast_rank=10&RID=X79P9D9H013) | [AZU99292.1](https://www.ncbi.nlm.nih.gov/protein/1549120201) | [AZU99284.1](https://www.ncbi.nlm.nih.gov/protein/1549120193) |  |
| Acinetobacter phage vB_AbaP_APK93 | [MK257721.1](https://www.ncbi.nlm.nih.gov/nucleotide/MK257721.1?report=genbank&log$=nucltop&blast_rank=12&RID=X77ND6AR016) | [AZU99342.1](https://www.ncbi.nlm.nih.gov/protein/1549120252) | [AZU99334.1](https://www.ncbi.nlm.nih.gov/protein/1549120244) |  |
| Acinetobacter phage vB_AbaP_PMK34 | [MN433707.1](https://www.ncbi.nlm.nih.gov/nucleotide/MN433707.1?report=genbank&log$=nucltop&blast_rank=6&RID=X77ND6AR016) | [QGF20174.1](https://www.ncbi.nlm.nih.gov/protein/1774198599) | [QGF20167.1](https://www.ncbi.nlm.nih.gov/protein/1774198592) | * |
| Acinetobacter phage vB_AbaP_APK44 | [MN604238.1](https://www.ncbi.nlm.nih.gov/nucleotide/MN604238.1?report=genbank&log$=nucltop&blast_rank=7&RID=X77ND6AR016) | [QGK90444.1](https://www.ncbi.nlm.nih.gov/protein/1776603771) | [QGK90436.1](https://www.ncbi.nlm.nih.gov/protein/1776603763) |  |
| Acinetobacter phage vB_AbaP_APK87 | [MN604239.1](https://www.ncbi.nlm.nih.gov/nucleotide/MN604239.1?report=genbank&log$=nucltop&blast_rank=18&RID=X77ND6AR016) | [QGK90498.1](https://www.ncbi.nlm.nih.gov/protein/1776603871) | [QGK90490.1](https://www.ncbi.nlm.nih.gov/protein/1776603863) |  |
| Acinetobacter phage vB_AbaP_APK89 | [MN651570.1](https://www.ncbi.nlm.nih.gov/nuccore/MN651570.1) | [QGK90394.1](https://www.ncbi.nlm.nih.gov/protein/1776603676) | [QGK90386.1](https://www.ncbi.nlm.nih.gov/protein/1776603668) |  |
| Acinetobacter virus vB_AbaP_AGC01 | [MT263719.1](https://www.ncbi.nlm.nih.gov/nucleotide/MT263719.1?report=genbank&log$=nucltop&blast_rank=3&RID=X77ND6AR016) | [QIW86364.1](https://www.ncbi.nlm.nih.gov/protein/QIW86364.1?report=genbank&log$=prottop&blast_rank=72&RID=X83N1PRF013) | [QIW86356.1](https://www.ncbi.nlm.nih.gov/protein/1829637936) | ** |
| Acinetobacter phage vB_AbaP_APK81 | [MT741944.1](https://www.ncbi.nlm.nih.gov/nucleotide/MT741944.1?report=genbank&log$=nucltop&blast_rank=4&RID=X77ND6AR016) | [QNO11418.1](https://www.ncbi.nlm.nih.gov/protein/1899069131) | [QNO11410.1](https://www.ncbi.nlm.nih.gov/protein/1899069123) |  |
| Acinetobacter phage Pipo | [MW366783.1](https://www.ncbi.nlm.nih.gov/nucleotide/MW366783.1?report=genbank&log$=nucltop&blast_rank=15&RID=X77ND6AR016) | [QQO92973.1](https://www.ncbi.nlm.nih.gov/protein/1955694163) | [QQO92965.1](https://www.ncbi.nlm.nih.gov/protein/1955694155) |  |
| Acinetobacter phage APK09 | [MZ868724.1](https://www.ncbi.nlm.nih.gov/nucleotide/MZ868724.1?report=genbank&log$=nucltop&blast_rank=5&RID=X79P9D9H013) | [UAW09804.1](https://www.ncbi.nlm.nih.gov/protein/2095891525) | [UAW09796.1](https://www.ncbi.nlm.nih.gov/protein/2095891517) |  |
| Acinetobacter phage APK77 | [MZ868726.1](https://www.ncbi.nlm.nih.gov/nucleotide/MZ868726.1?report=genbank&log$=nucltop&blast_rank=2&RID=X77ND6AR016) | [UAW09916.1](https://www.ncbi.nlm.nih.gov/protein/2095891584) | [UAW09908.1](https://www.ncbi.nlm.nih.gov/protein/2095891576) |  |
| Acinetobacter phage Abp1 | [NC_021316.1](https://www.ncbi.nlm.nih.gov/nucleotide/NC_021316.1?report=genbank&log$=nucltop&blast_rank=3&RID=X79P9D9H013) | [YP_008058239.1](https://www.ncbi.nlm.nih.gov/protein/YP_008058239.1?report=genbank&log$=prottop&blast_rank=18&RID=X7AJ41DD013) | [YP_008058231.1](https://www.ncbi.nlm.nih.gov/protein/509139338) | ** |
| Acinetobacter phage vB_AbaP_AS11 | [NC_041915.1](https://www.ncbi.nlm.nih.gov/nucleotide/NC_041915.1?report=genbank&log$=nucltop&blast_rank=13&RID=X77ND6AR016) | [YP_009599281.1](https://www.ncbi.nlm.nih.gov/protein/1631921407) | [YP_009599273.1](https://www.ncbi.nlm.nih.gov/protein/1631921399) |  |
| Acinetobacter phage vB_AbaP_B5 | [NC_042005.1](https://www.ncbi.nlm.nih.gov/nucleotide/NC_042005.1?report=genbank&log$=nucltop&blast_rank=16&RID=X77ND6AR016) | [YP_009610433.1](https://www.ncbi.nlm.nih.gov/protein/1631932664) | [YP_009610425.1](https://www.ncbi.nlm.nih.gov/protein/1631932656) | * |
| Acinetobacter phage vB_ApiP_P1 | [NC_042006.1](https://www.ncbi.nlm.nih.gov/nucleotide/NC_042006.1?report=genbank&log$=nucltop&blast_rank=5&RID=X77ND6AR016) | [YP_009610482.1](https://www.ncbi.nlm.nih.gov/protein/1631932714) | [YP_009610475.1](https://www.ncbi.nlm.nih.gov/protein/1631932707) | * |
| Acinetobacter phage vB_AbaP_B09_Aci08 | [NC_048081.1](https://www.ncbi.nlm.nih.gov/nucleotide/NC_048081.1?report=genbank&log$=nucltop&blast_rank=8&RID=X77ND6AR016) | [YP_009814060.1](https://www.ncbi.nlm.nih.gov/protein/1842026999) | [YP_009814052.1](https://www.ncbi.nlm.nih.gov/protein/1842027005) | ** |
| Acinetobacter phage vB_AbaP_PE21 | [OL964948.1](https://www.ncbi.nlm.nih.gov/nucleotide/OL964948.1?report=genbank&log$=nucltop&blast_rank=14&RID=X77ND6AR016) | [ULG00671.1](https://www.ncbi.nlm.nih.gov/protein/2196312701) | [ULG00663.1](https://www.ncbi.nlm.nih.gov/protein/2196312693) |  |
| Acinetobacter phage vB_Ab4_Hep4 | [OP019135.1](https://www.ncbi.nlm.nih.gov/nucleotide/OP019135.1?report=genbank&log$=nucltop&blast_rank=10&RID=X77ND6AR016) | [UVD33039.1](https://www.ncbi.nlm.nih.gov/protein/UVD33039.1?report=genbank&log$=prottop&blast_rank=31&RID=X83N1PRF013) | [UVD33047.1](https://www.ncbi.nlm.nih.gov/protein/2287970659) | * |
| Acinetobacter phage AbpL | [OP171942.1](https://www.ncbi.nlm.nih.gov/nucleotide/OP171942.1?report=genbank&log$=nucltop&blast_rank=17&RID=X77ND6AR016) | [UVD42134.1](https://www.ncbi.nlm.nih.gov/protein/UVD42134.1?report=genbank&log$=prottop&blast_rank=96&RID=X79MEV6B013) | [UVD42127.1](https://www.ncbi.nlm.nih.gov/protein/2287984964) | * |

BLASTn search made 30th of January 2023. The protein ids for the tailspike protein and the capsid protein were searched from the whole genome sequences. *Tailspike was reannotated based on the protein sequence homology, **Tailspike and capsid proteins were reannotated based on the protein sequence homology


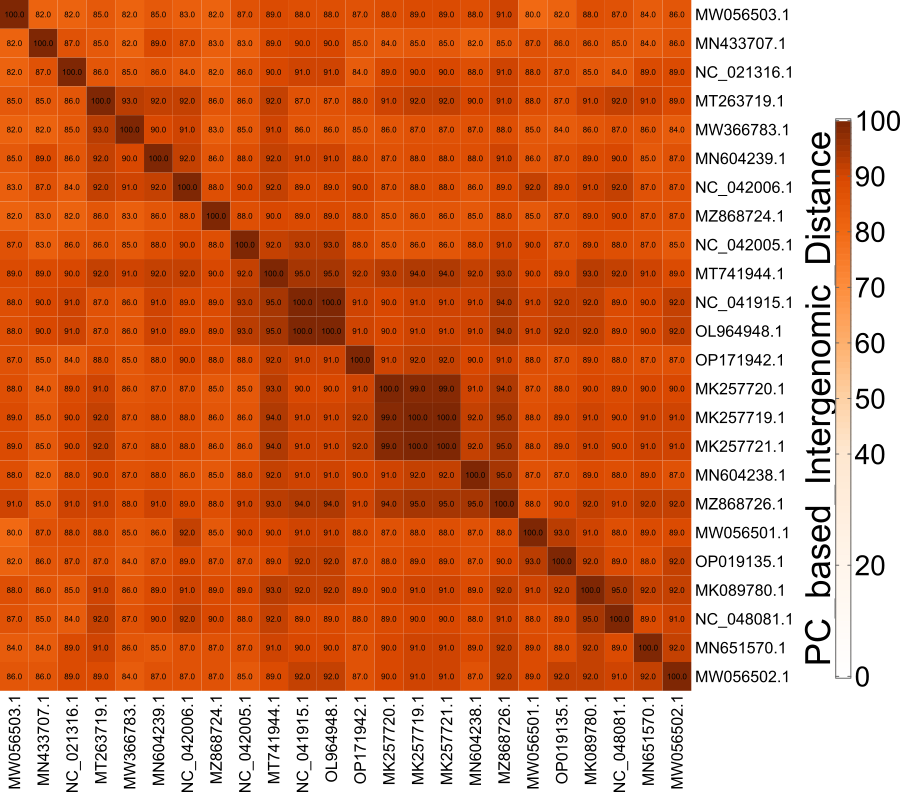


**Supplementary Fig.2.** Similarity heatmap of phage genomes used for phylogeny analysis. Accession numbers MW056501.1, MW056502.1, and MW056503.1 indicate phages fBenAci001, fBenAci002, and fBenAci003, respectively. Heatmap was generated with VirClust WEB server at Virus Intergenomic Distance Calculator VIRIDIC [1].

References

1. Moraru C, Varsani A, Kropinski AM (2020) VIRIDIC—A novel tool to calculate the intergenomic similarities of prokaryote-infecting viruses. Viruses 12:. https://doi.org/10.3390/v12111268
